# Gene Expression Dynamics at the Neurovascular Unit During Early Regeneration After Cerebral Ischemia/Reperfusion Injury in Mice

**DOI:** 10.3389/fnins.2020.00280

**Published:** 2020-04-02

**Authors:** Roxane-Isabelle Kestner, Franziska Mayser, Rajkumar Vutukuri, Lena Hansen, Stefan Günther, Robert Brunkhorst, Kavi Devraj, Waltraud Pfeilschifter

**Affiliations:** ^1^Department of Neurology, University Hospital Frankfurt, Goethe University, Frankfurt am Main, Germany; ^2^Department of General Pharmacology and Toxicology, Pharmazentrum Frankfurt, University Hospital Frankfurt, Goethe University, Frankfurt am Main, Germany; ^3^Department of Cardiac Development and Remodeling, Max Planck Institute for Heart and Lung Research, Bad Nauheim, Germany; ^4^Institute of Neurology (Edinger Institute), University Hospital Frankfurt, Goethe University, Frankfurt am Main, Germany

**Keywords:** stroke, blood-brain barrier, high-throughput nucleotide sequencing, matrix metalloproteinases, reperfusion injury, translational medical research

## Abstract

With increasing distribution of endovascular stroke therapies, transient middle cerebral artery occlusion (tMCAO) in mice now more than ever depicts a relevant patient population with recanalized M1 occlusion. In this case, the desired therapeutic effect of blood flow restauration is accompanied by breakdown of the blood-brain barrier (BBB) and secondary reperfusion injury. The aim of this study was to elucidate short and intermediate-term transcriptional patterns and the involved pathways covering the different cellular players at the neurovascular unit after transient large vessel occlusion. To achieve this, male C57Bl/6J mice were treated according to an intensive post-stroke care protocol after 60 min occlusion of the middle cerebral artery or sham surgery to allow a high survival rate. After 24 h or 7 days, RNA from microvessel fragments from the ipsilateral and the contralateral hemispheres was isolated and used for mRNA sequencing. Bioinformatic analyses allowed us to depict gene expression changes at two timepoints of neurovascular post-stroke injury and regeneration. We validated our dataset by quantitative real time PCR of BBB-associated targets with well-characterized post-stroke dynamics. Hence, this study provides a well-controlled transcriptome dataset of a translationally relevant mouse model 24 h and 7 days after stroke which might help to discover future therapeutic targets in cerebral ischemia/reperfusion injury.

## Introduction

Over the past decades, management of ischemic stroke has improved dramatically. The “penumbra” concept has given rise to pharmacological and mechanical recanalization therapies which are able to rescue ischemic brain tissue if applied early enough. Unfortunately, only a fraction of all stroke patients benefits from those therapies due to a narrow time window for optimal treatment and relevant contraindications. Additionally, after successful recanalization reperfusion-associated brain injury occurs due to secondary impairment of the blood-brain barrier (BBB) (Choi and Pile-Spellman, 2018). Despite intensive experimental research, promising drug candidates for acute and postacute stroke treatment have failed to translate into new therapeutic strategies (O’Collins et al., 2006). Better understanding of the processes at the BBB and their temporal dynamics is critical to overcome this “translational roadblock.”

Target of many of these experimental approaches is the neurovascular unit (NVU) which comprises brain capillaries as well as adjacent pericytes, astrocytes, along with their extracellular matrix (ECM) in addition to microglia and neurons (Iadecola, 2017). The specialized endothelium of those capillaries forms the BBB, a highly polarized and tightly regulated interface of the brain microvasculature and CNS tissue. The interplay of an unfenestrated network of tight junctions and specialized transporters allows a selective permeability to water and nutrients yet keeping toxins and pathogens out (Cummins, 2012; Knowland et al., 2014).

Ischemic strokes are caused by an acute occlusion of a cerebral vessel leading to oxygen and glucose deprivation in the dependent brain tissue. Due to a consecutive breakdown of the neuronal ionic balance release of glutamate and induction of glutamate-mediated pathways occurs and triggers cell death by massive calcium influx. This so-called glutamate excitotoxicity is one of the first phenomena to take place in the ischemic lesion leading to extensive cell death (Szydlowska and Tymianski, 2010). In parallel, lack of oxygen causes a failure of the respiratory chain and mitochondrial function so that pH is reduced and free radicals expand the local tissue damage (Lipton, 1999). Subsequently, a sterile inflammatory reaction going along with a hyperacute imbalance of matrix metallopeptidases (MMPs), especially MMP-9, and their endogenous counterplayers, tissue inhibitor of metallopeptidases (TIMP)-1 and -2 (Barr et al., 2010; Lenglet et al., 2014) triggers the destruction of the crucial endothelial-ECM interface of the BBB (Edwards and Bix, 2018). On the other hand, a step-wise degradation of endothelial cells in the oxygen-deprived area has been observed in the context of permanent and reperfused stroke even without disintegration of tight junction molecules (Krueger et al., 2015). Altogether, BBB function is disrupted and allows invasion of immune cells and pathogenic factors.

Unfortunately, re-establishment of blood-flow in the occluded vessel does not simply reverse those effects. MMPs stay upregulated after reperfusion and free radicals have been reported to even increase in this context as degraded lipid components serve as substrates for further production of reactive oxidative species (Bai and Lyden, 2015). In combination with a transient hyperemia which can last for hours or even days after recanalization treatment (Kidwell et al., 2001) patients are at risk for secondary vasogenic edema and hemorrhagic transformation of the infarcted area (Yang et al., 2007; Strbian et al., 2008; Sun et al., 2017). This so-called cerebral ischemia/reperfusion (I/R) injury hampers the clinical benefit of recanalization therapies and is target of experimental treatment strategies.

Previous experimental studies have shown a neuroprotective effect of acute MMP-9 inhibition in stroke (Asahi et al., 2000) whereas MMP-9 inhibition in the early recovery phase exacerbated stroke-induced brain damage (Zhao et al., 2006). Several other pathophysiological processes have shown a similar unexpected biphasic function after stroke (Ma et al., 2017; Sandvig et al., 2018). Only long-term observations of cohorts with acceptable survival rates allow to assess the full picture of post-ischemic vascular inflammation, its resolution and tissue repair.

We therefore performed a transcriptomic analysis of brain microvessels of a carefully monitored mouse population at 24 h and 7 days post transient middle cerebral artery occlusion (tMCAO) to depict an early timepoint after transient vessel occlusion, dominated by cytotoxic, protein degrading and inflammatory pathways as well as a later one, displaying first regenerative processes amid persisting immune cell infiltration and cytotoxic conditions. By this, we obtained comprehensive and unbiased insights on the complex molecular changes that occur at the BBB in the first days after cerebral I/R injury in mice.

## Materials and Methods

### Animals

A total of 95 male 10–12 weeks old C57Bl/6J mice (25–30 g) were purchased from Charles River Laboratories (Sulzfeld, Germany) and housed on a 12 h/12 h light/dark cycle with *ad libitum* access to food and water. Due to fight wounds, three mice were excluded before intervention. Experiments were performed in compliance with the ARRIVE guidelines for animal research, in accordance with the German Protection of Animals Act and the guidelines for care and use of laboratory animals by the local committee (Regierungspräsidium Darmstadt, approval number FU/1143).

### Transient Middle Cerebral Artery Occlusion and Post-stroke Care

**FIGURE 1:**
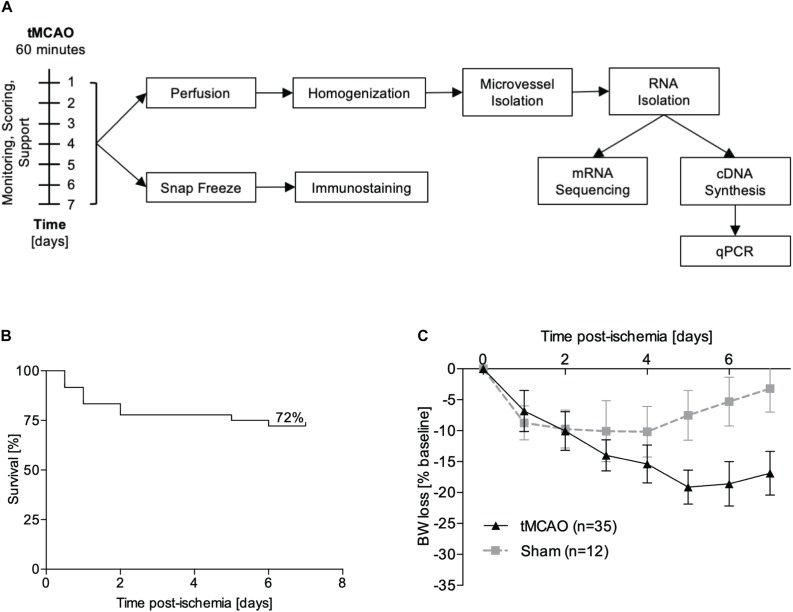
Experimental design. **(A)** Male C57Bl/6 mice underwent tMCAO or sham surgery and were supported for either 24 h or 7 days. For RNA sequencing, perfused microvessels from the ipsi- and contralateral hemispheres were isolated and pooled (*n* = 3 hemibrains per sample, *n* = 3 samples per group). For immunofluorescence staining, brains were snap frozen and cut into 10 μm sections. **(B)** Kaplan–Meier plot for survival of animals after tMCAO (*n* = 35). **(C)** Weight loss after sham (gray line, *n* = 12) and tMCAO surgery (black line, *n* = 35) presented as percentage reduction from preoperative body weight ± SD.

The right middle cerebral artery was occluded as described previously (Pfeilschifter et al., 2012). Briefly, mice were anesthetized with 1.5% isoflurane and kept on a temperature-controlled heating mat throughout surgeries. After ligation of the right common and external carotid arteries, a standard silicone rubber-coated monofilament (6-0 medium, Doccol, Sharon, MA, United States) was advanced until the branching point of the middle cerebral artery, allowing reperfusion after 60 min. In sham controls, the filament was retracted immediately after reaching the branching point. Analgesia was achieved by i.p. injections of buprenorphine (0.1 mg/kg bodyweight) 30 min before surgery and in 8 h-intervals during the first 48 h. Mice were housed in groups of 3–5 animals and cages kept on a heating mat (Beurer, Ulm, Germany). Daily monitoring included body weight, rectal temperature and clinical signs of pain, subarachnoid hemorrhage (SAH) or brain herniation. Experimental design is summarized in Figure 1A. On day 1, 3, and 7 after surgery, neurological and general deficits were assessed using the Experimental Stroke Scale (ESS) established by Lourbopoulos et al. (2017). Food and water were supplied adapted to the individual animal’s need according to the maximized post-stroke support protocol described there. This procedure included daily monitoring of weight and body temperature and, according to the hereby identified condition of the animals, oral feeding of 1–3 mL of dissolved food pellets per day. Liquids were provided by subcutaneous application of 2–4 mL warmed 0.9% saline given in two doses per day. Additionally, jelly food and food pellets were kept on the cage floor to be easily accessible for recovering individuals. Animals were sacrificed either 24 h or 7 days post-stroke. Stroke-related mortality was 12% in the 24 h- and 28% in the 7 day-cohort. Four mice were excluded from the study and calculation of stroke related mortality: three animals due to proof of artificial SAH in post-mortem autopsy and one sham-operated animal due to stroke symptoms.

### Isolation of Mouse Brain Microvessels

**FIGURE 2:**
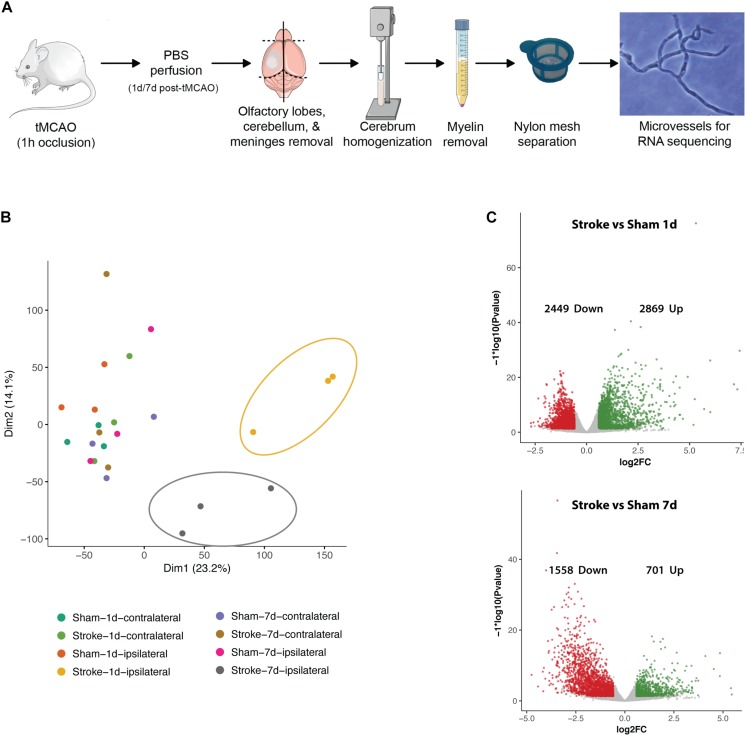
Basic characteristics post-stroke transcriptomics in microvessels. **(A)** Microvessel isolation protocol. **(B)** PCA plot of all samples. Distinct clusters are highlighted (yellow/upper right circle: ipsilateral hemispheres 24 h after tMCAO, gray/central circle: ipsilateral hemispheres 7 days after tMCAO). **(C)** Volcano plots of regulated genes in comparison 5 (stroke ipsilateral versus sham ipsilateral 24 h) and 6 (stroke ipsilateral versus sham ipsilateral 7 days). Transcripts accepted as regulated: mean > 5 counts, log_2_fc <> ±0.585, FDR ≤ 0.05.

Isolation of mouse brain microvessels (MBMVs) were isolated as indicated in Figure 2A using a previously described protocol (Gurnik et al., 2016). All steps were performed at 4°C or on ice. In brief, post anesthesia, mice were transcardially perfused with sterile phosphate buffered saline (PBS; Gibco, Thermo Fisher Scientific) for 10 min using a peristaltic pump (Ismatec, Cole-Parmer, Wertheim, Germany). Brains were macroscopically checked for broad cortical infarction and collected in microvessel buffer (MVB: 15 mM HEPES, 147 mM NaCl, 4 mM KCl, 3 mM CaCl_2_, 1.2 mM MgCl2, 5 mM glucose, and 0.5% BSA, pH 7.4) on ice. Individual stroke volumes could not be quantified during the isolation process without risking loss of vital material and artificial tissue reactions. Medium stroke sizes in our surgery setup covering about 60% of the hemisphere have been reported before (Friedländer et al., 2017). To estimate stroke volumes in the present study, male C57B/6J mice were operated by RK under same conditions as the mice used for mRNA sequencing. After 24 h, mice were sacrificed, brains removed carefully from the skull and cut into 1 mm sections. Subsequently, brain slices were stained in 2% 2,3,5-Triphenyl-tetrazolium chloride (TTC) at 37°C for 5 min and imaged on a flatbed color scanner (CanoScan LiDE 100; Canon, Tokyo, Japan). The hereby obtained Photoshop images were used for semi-automatical stroke volume calculation by an ImageJ macro established by Friedländer et al. (2017). Individual results are summarized in Supplementary Figure IV (mean stroke volume 57.2%, median 55.6%).

After removal of the cerebellum, brain stem and olfactory lobes, meninges were peeled off by rolling the cerebrum on autoclaved Whatman filter paper (Schleicher & Schuell, München, Germany). Hemispheres were separated and pooled (from three animals). Tissue was homogenized in MVB using a Dounce homogenizer (Wheaton, 0.025 mm clearance) attached to an electrical overhead stirrer (2000 rpm, VOS 14, VWR) and centrifuged at 400 × *g* for 10 min. Myelin fat was separated by resuspension of the pellet in 25% BSA and centrifugation at 2000 × *g* for 30 min. Subsequently, the cell pellet was resuspended in MVB and filtered through a 100 μm cell strainer (Falcon, Corning, NY, United States) to remove large vessel fragments and tissue clusters. The flow-through was filtered through a 40 μm cell strainer (Falcon, Corning, NY, United States) to be cleared from erythrocytes, dead cell nuclei and cell debris. Microvessels were harvested directly from the 40 μm mesh in RLT buffer (Qiagen, Düsseldorf, Germany) containing Dithiothreitol (DTT, 40 mM; Roche Diagnostics, Indianapolis, IN, United States) and stored at −80°C until RNA isolation. Successful isolation of microvessel fragments was verified by light microscopy assessment.

A total of 32 MBMV samples were generated and included into 8 groups (sham ipsilateral 24 h, sham contralateral 24 h, tMCAO ipsilateral 24 h, tMCAO contralateral 24 h, sham ipsilateral 7 days, sham contralateral 7 days, tMCAO ipsilateral 7 days, tMCAO contralateral 7 days). One group consisted of *n* = 4 samples each. One sample was generated by pooling three hemispheres of the same kind after brain removal and separation of ipsi- and contralateral hemispheres. Analysis identified four samples as outliers and further analysis was performed on *n* = 3 samples per group. In total, one group contains brain hemispheres of 3 × 3 = 9 animals. The work flow and experimental design is shown in Figure 1A.

### mRNA Sequencing From MBMVs

RNA was extracted from MBMVs using Qiagen RNeasy plus Micro Kit (Qiagen, Düsseldorf, Germany) following the instructions provided by the manufacturer. RNA concentration measured by Qubit fluorometer (Thermo Fisher Scientific) did not differ significantly within the samples [mean 24 h 22.24 (ng/μL) ± SD 4.55 (ng/μL), mean 7 d 27.82 (ng/μL) ± SD 2.51 (ng/μL), mean total 25.03 (ng/μL) ± SD 9.05 (ng/μL)]. Samples were deep frozen (−80°C) and transferred to the mRNA sequencing facility at Max Planck Institute for Heart and Lung Research Bad Nauheim.

After verification of RNA quality (LabChip Gx Touch 24, Perkin Elmer), equal amounts of RNA from each sample were subjected to mRNA sequencing using a NextSeq 500 sequencer (Illumina, San Diego, CA, United States). FastQC tool (Babraham Bioinformatics, 2010) was used to verify signal quality, adaptor content and duplication rate. Adapters were removed and data trimmed; reads of a length between 30 and 150 base pairs were accepted for further analysis. Using STAR 2.4.0a, results were aligned to the murine mm10 genome (Ensembl) with maximum ratio of mismatches to mapped length set to 10%. Reads matching multiple genes or regions were excluded.

### Quantitative Real Time PCR

Part of the total RNA (40 ng) from each sample was subjected to cDNA synthesis using RevertAid Reverse Transcriptase Kit (Thermo Fisher Scientific, Darmstadt, Germany). Quantitative Real Time PCR (qRT-PCR) was performed using TaqMan Gene Expression Assays in Applied Biosystems 96-well PCR plates (Thermo Fisher Scientific). Plates were sealed with Clear Seal Diamond Heat Sealing Film (Thermo Fisher Scientific) and run in an AB7500 fast system (Applied Biosystems, Thermo Fisher Scientific, Foster City, CA, United States). TaqMan probes (Thermo Fisher Scientific) were used to quantify transcripts of hypoxanthine phosphoribosyltransferase 1 (HPRT, Mm03024075_m1), matrix metalloproteinase 9 (MMP9, Mm00442991_m1) and tissue inhibitor of metalloproteinases 1 (TIMP1, Mm01341361_m1).

### Immunohistochemical Staining on Cryosections

After sacrificing the animals, brains were extracted and natively frozen in Tissue TEK O.C.T. Compound (Sakura Finetek, Torrance, CA, United States) on dry ice and stored at −80°C. Coronal 10 μm sections were fixed in 4% PFA for 10 min and blocked in 10% fetal bovine serum in TBS-TritonX 0.3%. Tissue was incubated with primary antibodies (mouse-anti-mouse occludin, Invitrogen/Thermo Fisher Scientific #OC-3F10, 1:200; rat-anti-mouse CD31, BD Biosciences #BD553370, 1:200) for 2 h at room temperature followed by 1 h-incubation using fluorescence-conjugated secondary antibodies (donkey-anti-rat Alexa Fluor 488, donkey-antimouse Alexa Fluor 568, 1:400). After counter-staining for nucleic acids with 4’,6-diamidino-2-phenylindole (DAPI, Sigma-Aldrich, St. Louis, MO, United States, 1:1000) sections were mounted with fluorescent mounting medium (Dako, Agilent, Santa Clara, CA, United States). Images were acquired at 40× magnification with a Nikon 80i microscope and quantified using NIS elements software (Nikon, Düsseldorf, Germany). Exposure time and LUTs settings were maintained at same values for all samples.

### Statistical Methods

Reads obtained by *mRNA sequencing* were normalized and differentially analyzed using DESeq2 package version 1.14.1 (Love et al., 2014). To define genes as significantly regulated, a logarithmic fold change of at least ± 0.585, a mean value higher than 5 and *p*-value lower than 0.05 adjusted by Benjamini–Hochberg procedure for multiple comparisons was required.

For *qRT-PCR* validation cDNA levels were normalized to HPRT as this gene was identified as being unchanged between samples from ischemic and non-ischemic hemispheres. Differential expression was calculated by the 2^–ΔCT^ method. Assuming a normally distributed population we used a *t*-test for separate comparison of sample pairs (sham ipsilateral 24 h versus tMCAO ipsilateral 24 h, sham ipsilateral 7 days versus tMCAO ipsilateral 7 days). A *p*-value below 0.05 was accepted as significant.

For *IHC* validation on cryosections we normalized occludin staining to CD31 positive vessels. To achieve this, we defined binaries for both antibodies by signal intensity in NIS elements software and measured the respective areas were measured. Afterward, the percentage of the CD31 + occludin-positive fraction from the total CD31-positive area was calculated. Separate comparison of sample pairs (core ipsilateral versus periinfarct ipsilateral 24 h, core ipsilateral versus core contralateral 24 h, core ipsilateral versus periinfarct ipsilateral 7 days, core ipsilateral versus core contralateral 7 days), was performed by *t*-test. A *p*-value below 0.05 was accepted as significant.

## Results

### Establishment of the Post-stroke Care Protocol

Using the post-stroke care protocol of Lourbopoulos et al. (2017) that we established in our facility, we achieved similar survival rates in the tMCAO model as previously reported. With optimized monitoring and feeding support, the mortality at 7 days post-stroke was reduced to 28% (Figure 1B) from an expected mortality of at least 59%. The weight loss curve reached the nadir at day 3 for sham operated mice and at day 5 for animals which underwent tMCAO. From day 6 onward, weight either stabilized or started to recover (Figure 1C).

### Expression Dynamics After Cerebral I/R Injury

Of a total of 141,136 reads in protein coding sequences 12,690 show significant regulation in altogether 7 comparisons (1: stroke ipsi- versus contralateral 24 h, 2: stroke ipsi- versus contralateral 7 days, 3: sham ipsi- versus contralateral 24 h, 4: sham ipsi- versus contralateral 7 days, 5: stroke ipsilateral versus sham ipsilateral 24 h, 6: stroke ipsilateral versus sham ipsilateral 7 days, and 7: stroke ipsilateral 24 h versus 7 days). Principal component analysis (PCA) revealed that all control samples (contralateral hemispheres of mice 24 h and 7 days post-stroke as well as both hemispheres of sham operated animals) cluster together. As no major differences were seen between the different controls we decided to continue analysis on comparisons 5–7. Heatmaps and pathway analyses of comparisons 1 and 2 can be seen in Supplementary Figure I. The ipsilateral hemispheres 24 h post-stroke form a distinctive cluster farthest from the controls. Ipsilateral hemispheres 7 days post-stroke cluster between the acutely ischemic tissue and the controls indicating post-stroke recovery (Figure 2B). Volcano plots (significance versus fold-change) show 2869 up- and 2449 downregulated genes in the ipsilateral hemisphere of tMCAO- compared to sham-operated animals 24 h post-surgery and 701 genes up- and 1558 downregulated 7 days post-surgery (Figure 2C).

**FIGURE 3:**
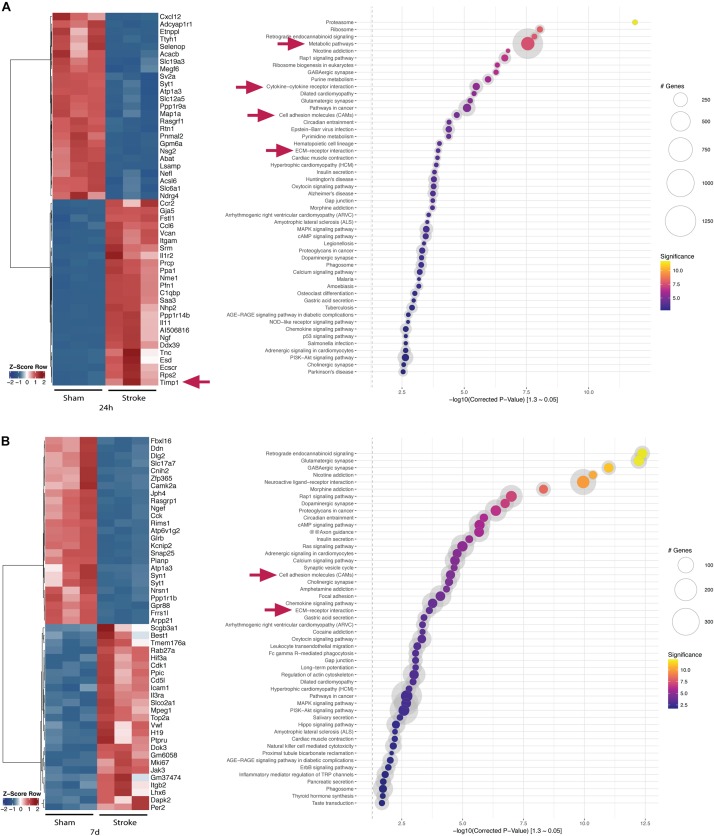
Top-regulated genes and pathways in the acute and postacute stage post-stroke after DESEQ2 normalization. **(A)** *Z*-score of top 25 up- and top 25 downregulated genes in the ipsilateral hemisphere of tMCAO- versus sham-operated animals 24 h post-surgery and gene set enrichment within the identified genes. **(B)** Z-score of top 25 up- and top 25 downregulated genes in the ipsilateral hemisphere of tMCAO- versus sham-operated animals 7 days post-surgery and gene set enrichment within the identified genes. Transcripts accepted as regulated: mean > 5 counts, log_2_fc <> ±0.585, FDR ≤ 0.05. Exemplary genes and gene sets are highlighted (arrows).

Figure 3 shows top 25 up- and downregulated genes at 24 h and 7 days post-stroke and their bioinformatic pathway analyses using KOBAS (Xie et al., 2011). Several genes belonging to the ECM-receptor interaction such as TIMP1 and cell adhesion molecules are dysregulated at 24 h post-stroke indicating an acute BBB breakdown. Metabolic and inflammatory pathways are highly regulated indicating the ischemic injury and the ensuing immune response (Figure 3A). At day 7, cell adhesion molecules and ECM-receptor interaction pathways are still dysregulated compared to sham control indicating BBB impairment also in the postacute phase.

### Quantitative Validation of Transcriptional Changes in Proteinases

**FIGURE 4:**
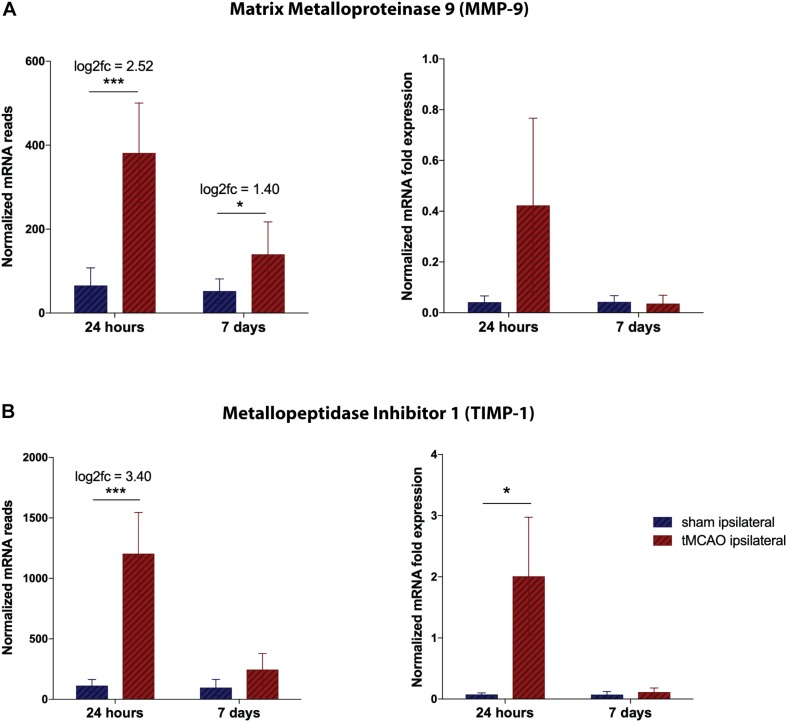
Validation of RNA sequencing dataset by qRT-PCR for proteinases at the NVU. **(A)** Total mRNA reads for TIMP-1 and MMP-9, normalized by DESEQ2. Presented as mean counts per group ± SD, logarithmic fold change and summarized *p*-value (*n* = 3 per group). **(B)** Quantitative validation by qPCR on remaining RNA and freshly isolated samples, normalized to HPRT. Presented as mean 2^–ΔCT^ values per group ± SD and summarized *p*-value (*n* = 3 per group). *p-value < 0.05, ***p-value < 0.0005.

As a measure of representativeness of the observed dynamics, we aimed to validate the well characterized post-ischemic changes in the MMP/TIMP system. MMP9 expression is dramatically increased after 24 h and still significantly upregulated after 7 days when compared to sham animals. qPCR measurements confirmed the acute overshoot of MMP9 (Figure 4A). Similarly, for TIMP1 a massive increase could be detected by RNA sequencing and reproduced by qPCR validation (Figure 4B).

Changes in MMP/TIMP expression are paralleled by breakdown of tight junction markers. By immunohistochemical staining (IHC), occludin signal was reduced at both time points within the stroke region in comparison to CD31 staining reaching significance on 7 days post-stroke. Representative images of the stroke core and corresponding area in the contralateral hemisphere as well as quantification of occludin staining fraction of CD31 positive vessels are shown in Supplementary Figure II.

### Expression Dynamics in the Early Regeneration Phase Post-stroke

**FIGURE 5:**
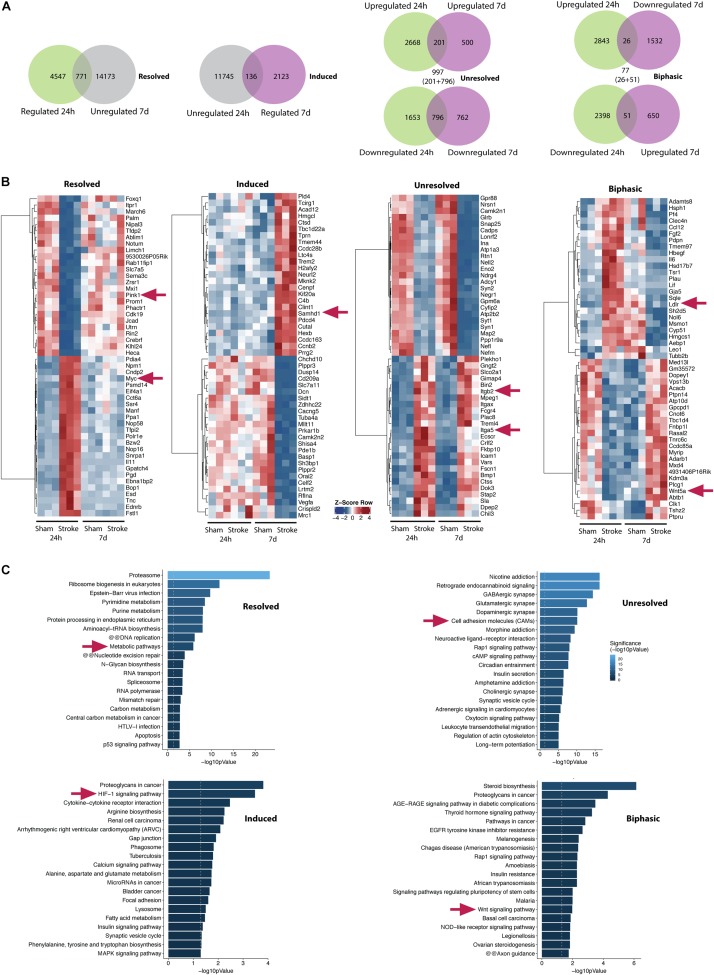
Transcriptomic dynamics at the NVU within one week after stroke. **(A)** Venn diagrams show numbers of genes which are regulated 24 h (first from left, green/left circle) and genes which are not regulated 7 days post-stroke in the ipsilateral hemisphere (first from left, gray/right circle). A total of 771 genes belonging to both groups are defined as resolved (first from left, overlap). A total of 136 genes (second from left, overlap) which are regulated 7 days (second from left, violet/right circle) and not regulated 24 h post-stroke (second from left, gray/left circle) are defined as induced. A total of 997 genes (second from right, sum of overlapping genes) which are either up- or downregulated at both time points are defined as unresolved. A total of 77 genes (first from right, sum of overlapping genes) which are oppositely regulated at both time points are defined as biphasic. **(B)** *Z*-score of top 25 up- and top 25 downregulated genes of induced, resolved and unresolved genes. For biphasic genes top 25 toward each direction are shown (first half: upregulated 24 h and downregulated 7 days post-stroke, second half: downregulated 24 h and upregulated 7 days post-stroke). **(C)** Corresponding bar graphs summarize gene set enrichment within the identified genes. Exemplary genes and gene sets are highlighted (arrows).

In order to characterize the intermediate-term expression dynamics post-stroke, we compared the gene expression pattern relative to sham controls at 24 h post-stroke with that of 7 days post-stroke. We defined four groups, namely resolved, induced, unresolved, and biphasic for further analyses as shown in the Venn diagrams of Figure 5A. Genes that are regulated at 24 h but not anymore at 7 days post-stroke belong to the resolved group (771 genes). Genes that are not regulated at 24 h but only at 7 days post-stroke belong to the induced group (131 genes). Genes that are upregulated at both time points (201 genes) along with genes that are downregulated at both time points (796 genes) belong to the unresolved group (201 + 796 = 997 genes). Finally, genes that are upregulated at 24 h but downregulated at 7 days (26 genes) and vice-versa (51 genes) are categorized into the biphasic group (26 + 51 = 77 genes).

Top 25 regulated genes in each direction for all the groups are summarized by heatmaps (Figure 5B). Top 20 pathways identified by gene set enrichment analysis (KOBAS KEGG) are shown in Figure 5C for all the groups. Several transcripts belonging to metabolic pathways such as PINK1 are resolved at day 7. This is accompanied by induction of pathways of angiogenic/ECM-remodeling (e.g., HIF-1- and proteoglycan-associated pathways) and inflammation at day 7 including genes of innate immunity (e.g., SAMHD1). However, some genes are unresolved by 7 days post-stroke comprising primarily cell adhesion and ECM-endothelial interaction molecules such as ITGB2, ITGA5 indicating persistent disruption of the BBB but also glutamatergic pathways as a hint for ongoing cytotoxicity.

The biphasic group represents genes that are counter-regulated by 7 days compared to 24 h post-stroke potentially in a compensatory manner for neurovascular regeneration. Wnt signaling is one of the major pathways regulated in such a biphasic manner at the NVU (Figure 5C). Wnt family members 5A (WNT5a) and 11 (WNT11) are significantly decreased 24 h and strongly induced 7 days after tMCAO. This is paralleled by induction of transcription factor SRY-Box 17 (SOX17) on day 7. Transcripts of LDL receptor related proteins 5 (LRP5) and 6 (LRP 6) which represent direct actors in the Wnt/β-catenin signaling cascade are acutely reduced (Supplementary Figure III).

## Discussion

The struggle for translation from bench to bedside in preclinical stroke research was paralleled by significant clinical advances that occurred rather independently. To date, patients benefit from effective recanalization therapies (Berkhemer et al., 2015; Lees et al., 2016) and the stroke unit concept which includes close monitoring, treatment of complications and rehabilitation (Indredavik et al., 1991; Stroke Unit Trialists’ Collaboration, 2013). Those innovations have changed stroke patients’ populations and needs while post-stroke care for mice has not been adapted accordingly.

Brain reperfusion injury after vessel occlusion and recanalization is best modeled by tMCAO but should go along with understanding and careful consideration of surgery-associated side effects. In a recent study, Lourbopoulos et al. (2017) set out to overcome technical confounders and mortality bias of this model by establishing a stroke unit-like post-stroke care protocol. They achieved a relevant increase in survival rates making this model now practicable for long-term observations. From our point of view, this is a fine example of translation from the clinic to the laboratory to make experimental models more reliable which may in turn facilitate translation from the laboratory to the clinic.

Since we are interested in time-dependent transcriptome changes at the NVU to identify potential therapeutic targets beyond the narrow time window of current recanalization-focused interventions, we chose this model to obtain a translationally relevant sample. The recorded transcriptomic dynamics representatively reflect inflammatory response at the brain microvasculature at an early and a late timepoint, its imbalance of cell adhesion and matrix components, the associated messengers and enzymes and the take-over by regenerative processes. PCA revealed that most dramatic transcriptomic changes occur at 24 h after stroke and already begin to resolve after 1 week. As acute I/R injury-associated breakdown of the BBB, leading to enhanced vascular edema and risk of hemorrhagic transformation gained increasing importance in the light of current stroke treatment, the focus of our study was on the brain microvasculature. Targeting the cellular and extracellular interplay within this compartment might help to overcome present therapeutic limitations (Gurnik et al., 2016; Manley et al., 2000). Exemplarily and as a validation of our dataset, we analyzed MMP-9 and TIMP-1 by quantitative PCR and could confirm the previously observed dramatic imbalance of MMPs and their inhibitors (Lenglet et al., 2014) in our samples.

In order to identify central regulatory pathways that emerge within the first week post-stroke at the BBB, we compared the gene expression patterns at 24 h and 7 days. Gene expression and bioinformatic pathway analyses indicate resolution of several pathways including metabolic and apoptotic signaling cascades by 7 days while – rather surprisingly – glutamatergic pathways remain unresolved (indicated by KEGG pathway terms “glutamatergic synapse” but also “nicotine addiction” and “morphine addiction”) (Kegg Pathway Maps, 2020). Late induction of PINK1, a mitophagy regulator to clear damaged mitochondria, indicates potential resolution of mitochondrial dysfunction and repair of I/R injury as shown previously in cardiac and renal ischemia (Siddall et al., 2013; Tang et al., 2018). The role of mitophagy in ischemic stroke is however not fully understood (Guan et al., 2018).

Furthermore, induction of HIF-1- and proteoglycan-associated pathways at 7 days indicate angiogenic remodeling. Mutations in SAMHD1, a dNTPase known to be important for innate immunity, have been shown to lead to cerebral vasculopathy and early onset stroke (Xin et al., 2011). Along this line, our dataset shows an induction of SAMHD1 in the postacute phase suggesting a potential role for SAMHD1 in post-stroke vascular repair and regeneration. However, several genes are unresolved 7 days after tMCAO especially cell-adhesion molecules such as integrins ITGB2 and ITGA5 as reported previously to be dysregulated in the acute phase (Edwards and Bix, 2018). Previous studies support the therapeutic significance of this group for integrin-based therapeutics (Ley et al., 2016).

A unique feature achieved by intermediate-term observation after stroke is the identification of biphasically regulated genes which might otherwise be misinterpreted. These genes are regulated in one direction at 24 h and in the opposite direction at 7 days post-stroke and potentially represent compensatory mechanisms essential for post-stroke recovery. As an intriguing example, actors of the Wnt signaling cascade, namely WNT5a, WNT11 and – as a downstream target of canonical Wnt signaling – SOX17 show such an expression pattern. While these factors and their direct responders LRP5 and LRP6 are significantly reduced in the acute phase, they show an induction 7 days after tMCAO. This finding is of special interest as Wnt/β-catenin signaling has recently been shown to conduct its crucial role in brain angiogenesis and BBB formation at the NVU (Liebner et al., 2008; Reis et al., 2012) potentially via SOX17 induction (Corada et al., 2018). Furthermore, pharmacological induction of β-Catenin by CHIR99021 was able to reactivate matured cardiomyocytes and therefore suggested as a future approach for regeneration after myocardial infarction (Fan et al., 2018). The same compound has been reported to suppress MMP9 release from macrophages *in vitro* (Kim et al., 2015) which also implies a potential use in BBB protection after cerebral I/R injury. Our current results might help in designing future *in vivo* assessment of this approach according to the biphasic regulation of the Wnt signaling cascade.

Previous RNA sequencing approaches after cerebral I/R injury used either the complete tissue of the affected hemisphere or focused on a specific cell type. Recently, a genome-wide analysis of brain tissue of rats 4.5 and 24 h post tMCAO depicted the acute and hyperacute transcriptional changes within the subcortex of the stroke hemisphere (Dergunova et al., 2018). They identified transcription factors, cytokines and hormones as being regulated already within the first hours after stroke onset and a shift within the expressed cytokine classes until 24 h post-stroke. KEGG pathway analysis showed some common regulations with our dataset e.g., in calcium signaling, MAPK signaling, and ECM-receptor interaction. Interestingly, oxytocine signaling was found to be regulated already at 4.5 h post-stroke which our analysis of microvessels after tMCAO in mice revealed to be unresolved over the first 7 days (Figure 5C). In another study by Duan et al. (2019) the affected hemisphere of rats was isolated 4 days after tMCAO and used to identify long non-coding (lnc)RNA transcripts as potential regulators of apoptosis-associated gene expression. They suggest important lncRNA to be co-expressed in KEGG pathway analysis with calcium signaling, TNF and EBV signaling which mainly comprises pro-inflammatory cytokines as well as PI3K-Akt signaling. Our dataset adds the prolonged scope of transcription dynamics with an explicit focus on the NVU including all its cellular compartments which is why in our study BBB-associated gene transcripts are much more prominent than in the aforementioned works.

Isolating microvessel fragments allows us to picture the dynamics in most components of the NVU (mainly endothelial cells, but to a lesser extent also pericytes, astrocytic endfeet, adjacent microglia and neurons). But it also goes along with certain limitations as it does not allow to pinpoint the specific cellular source of the observed changes. To keep contamination by peripheral blood cells to a minimum, all mice underwent thorough transcardial perfusion before brain isolation. Nevertheless, some immune cells might have been trapped in the microvasculature. From our point of view, this is not necessarily a weakness as immune cells play a crucial role in the vascular pathophysiology after I/R injury and contribute to the transcriptional pattern including cytokine-cytokine receptor interactions and expression of cell adhesion molecules. The contralateral hemispheres of the affected mice and both hemispheres of sham operated animals separately served as controls for stroke-associated changes.

Due to the hereby-caused complexity of the dataset our study is limited to a standard ischemia duration of 60 min and two investigated timepoints. Future studies are needed to investigate (a) shorter occlusion times to capture processes in primarily subcortical/minor strokes, (b) permanent filament insertion to assess differential dynamics between cerebral ischemia with and without reperfusion injury, (c) earlier (e.g., 72 h post-stroke, focusing on first- versus second-line inflammatory reaction) and later timepoints (e.g., 1 month after stroke to assess chronic repair processes), and (d) gene expression data from complete hemispheric tissue in relation to data specifically derived from microvessels of the ischemic hemisphere. Another limitation of our approach is that we isolated microvessels from the whole hemispheres as otherwise the material yield would have been too low for representative mRNA sequencing analysis. Therefore, it has to be kept in mind that our results stem from infarcted, peri-infarct and partly from intact brain regions. By this, genes of interest have to be validated subsequently on the spatial level. Exemplarily, we chose immunofluorescence staining to assess the differential expression of occludin in ischemic and non-ischemic vasculature. We found that within the infarct area, occludin expression was still significantly downregulated after 7 days while staining was normalized in the periinfarct cortex at this time point, in accordance with previous reports (Sun et al., 2017).

## Conclusion

In summary, we provide a well-controlled transcriptome dataset of a translationally relevant mouse cohort after tMCAO. Keeping the above-mentioned limitations in mind, this dataset may help to identify future therapeutic targets in post-stroke inflammation and repair processes including acute BBB alterations, glial scar formation, angiogenesis and neuroregeneration.

## Data Availability Statement

The authors declare that all supporting data are available within the article and Supplementary Material. The RNA sequencing dataset has been uploaded to GEO (record GSE131193).

## Ethics Statement

The animal study was reviewed and approved by the Regierungspräsidium Darmstadt.

## Author Contributions

RB, WP, and KD conceived of the presented idea and developed the experimental design together with R-IK. R-IK carried out the surgeries, post-stroke survival care, microvessel and mRNA isolation under support of RV, LH, and KD. SG performed the mRNA sequencing and supplied the respective graphs upon joined the data analysis with R-IK, FM, KD, WP, and RB. R-IK and FM were carried out the staining and qPCR validation. R-IK, WP, and KD prepared the manuscript.

## Conflict of Interest

The authors declare that the research was conducted in the absence of any commercial or financial relationships that could be construed as a potential conflict of interest.
