# Supplementary material for: Gene Expression Dynamics at the Neurovascular Unit During Early Regeneration After Cerebral Ischemia/Reperfusion Injury in Mice
Source: Front Neurosci. 2020 Apr 2;14:280. doi: 10.3389/fnins.2020.00280 (PMC7142359; doi:10.3389/fnins.2020.00280)
Supplement: Supplementary file 1 [file Data_Sheet_1.pdf]

SUPPLEMENTAL MATERIAL

Gene Expression Dynamics at the Neurovascular Unit During Early Regeneration After Cerebral Ischemia/Reperfusion Injury in Mice

Figure I

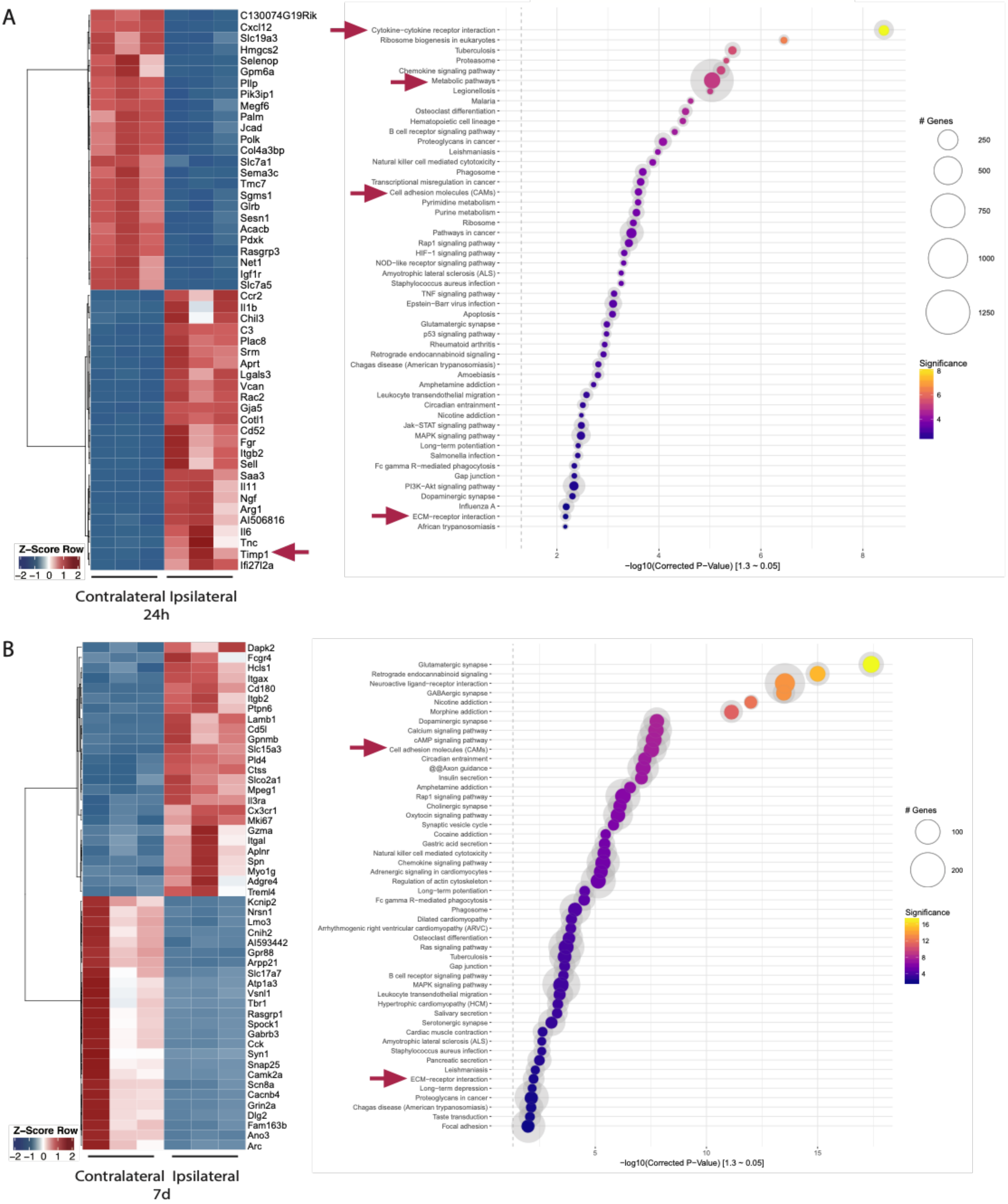

Top-regulated genes and pathways between ipsi- and contralateral post-stroke after DESEQ2 normalization. **A**: Z-Score of top 25 up- and top 25 downregulated genes in the ipsi- versus contralateral hemisphere of tMCAO-operated animals 24h post-surgery and gene set enrichment within the identified genes. **B**: Z-Score of top 25 up- and top 25 downregulated genes in the ipsi- versus contralateral hemisphere of tMCAO-operated animals 7d post-surgery and gene set enrichment within the identified genes. Transcripts accepted as regulated: mean > 5 counts, log2fc <>± 0.585, FDR ≤ 0.05. Exemplary genes and gene sets are highlighted (red arrows).

Figure II

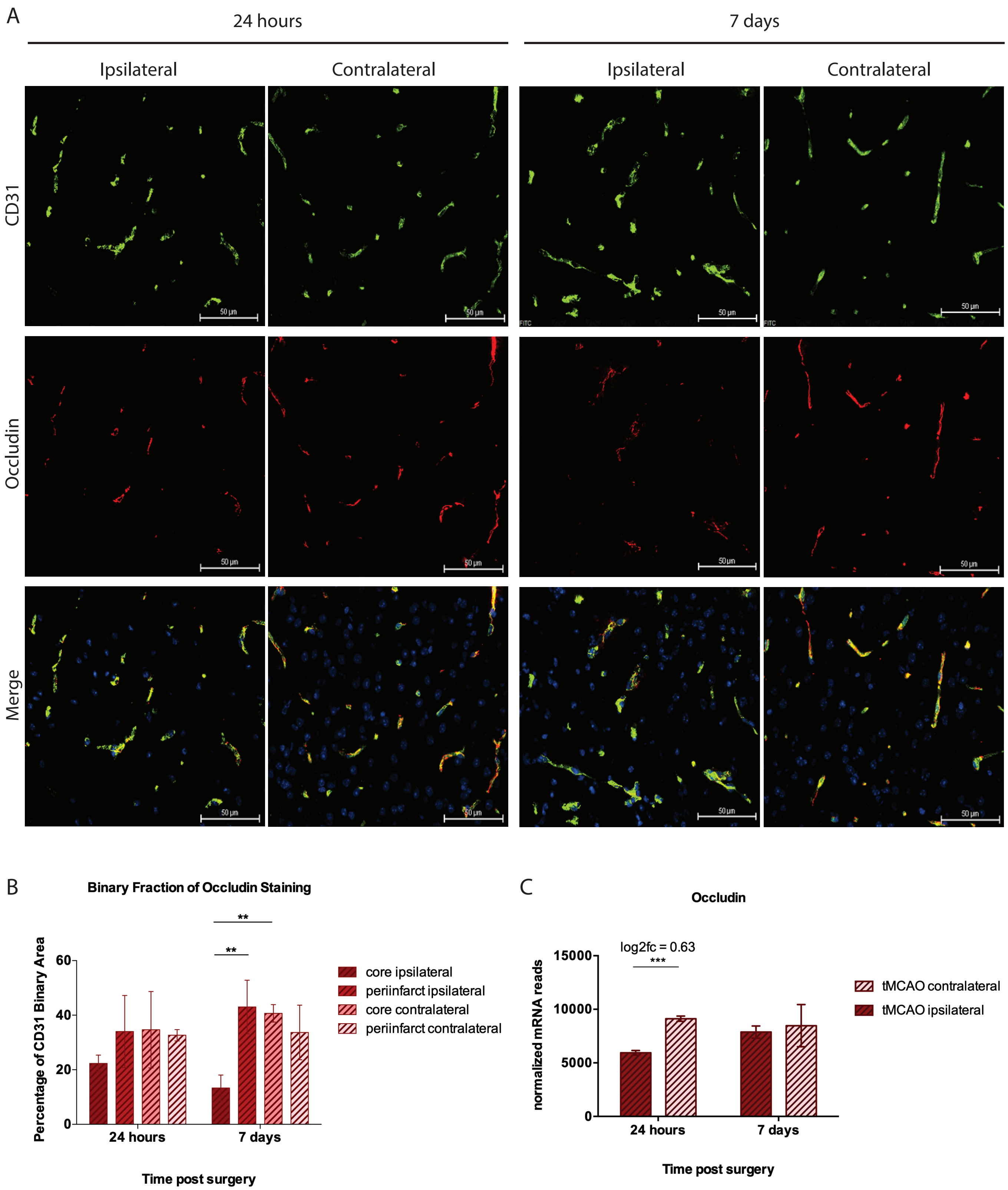

Time course of Occludin reduction at the NVU

A: Representative images of the stroke core and corresponding region on the contralateral hemisphere (green: CD31, red: Occludin, blue: DAPI), 40x magnification. **B**: Quantitative validation on protein level by immunofluorescence staining on brain cryosections 24h and 7d after tMCAO. Presented as mean percentage of Occludin vs. CD31 staining binaries ± SD at ipsi- and contralateral hemisphere in stroke core vs. periinfarct cortex. N = 3 per group. C: Total mRNA reads for OCLN, normalized by DESEQ2. Presented as mean counts per group ± SD, logarithmic fold change and summarized p-value (n = 4 per group).

Figure III

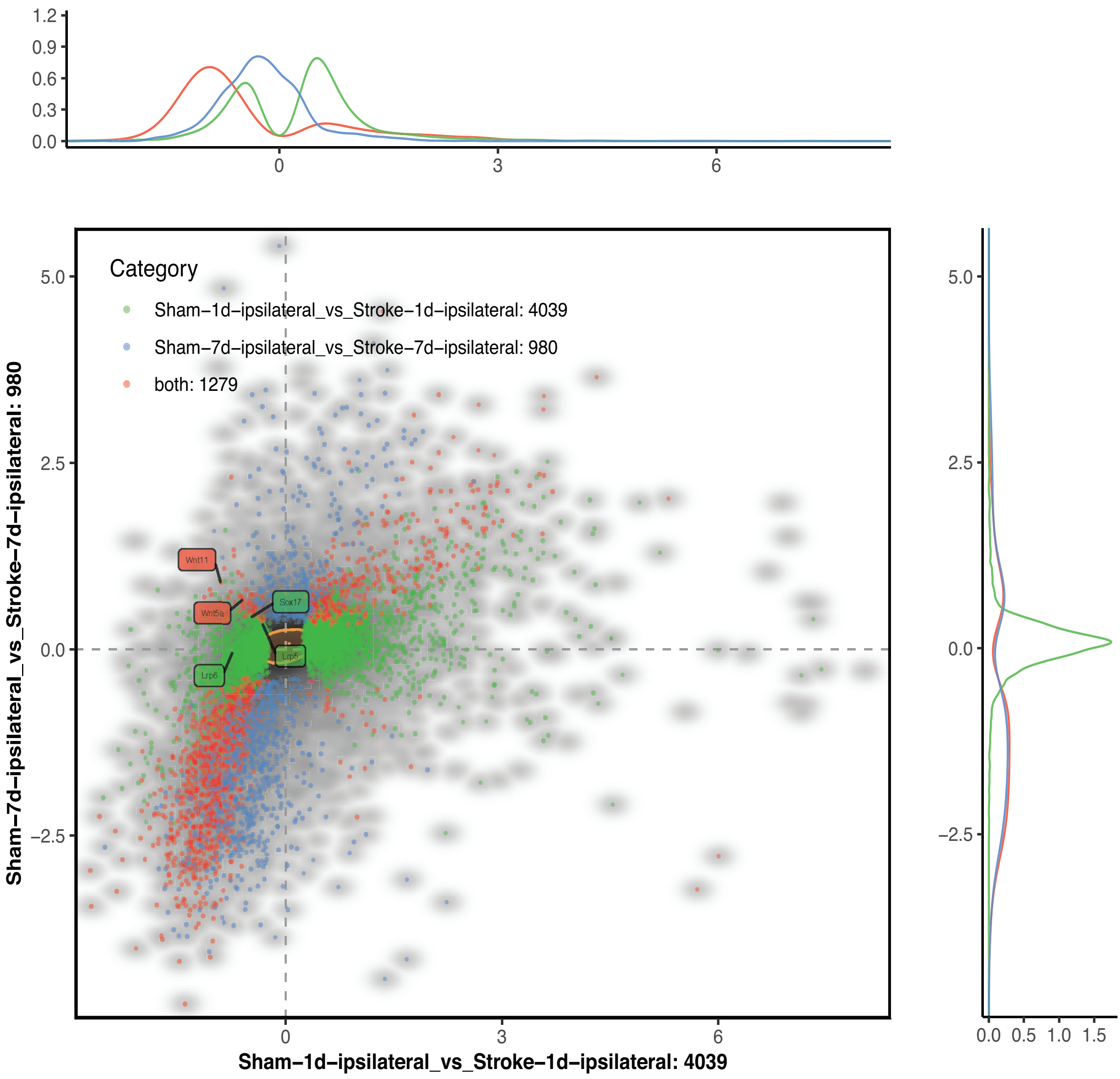

Global transcriptomic dynamics at the NVU within one week after stroke

Bagplot of regulated genes in comparison 5 (stroke ipsilateral versus sham ipsilateral 24h; x-axis) versus comparison 6 (stroke ipsilateral versus sham ipsilateral 7d, y-axis). Annotation of representative genes which are significantly reduced 24h and significantly increased 7d after tMCAO (red dots in upper left quadrant) or significantly downregulated 24h and non-significantly increased 7d after tMCAO (green dots in upper left quadrant).

Figure IV

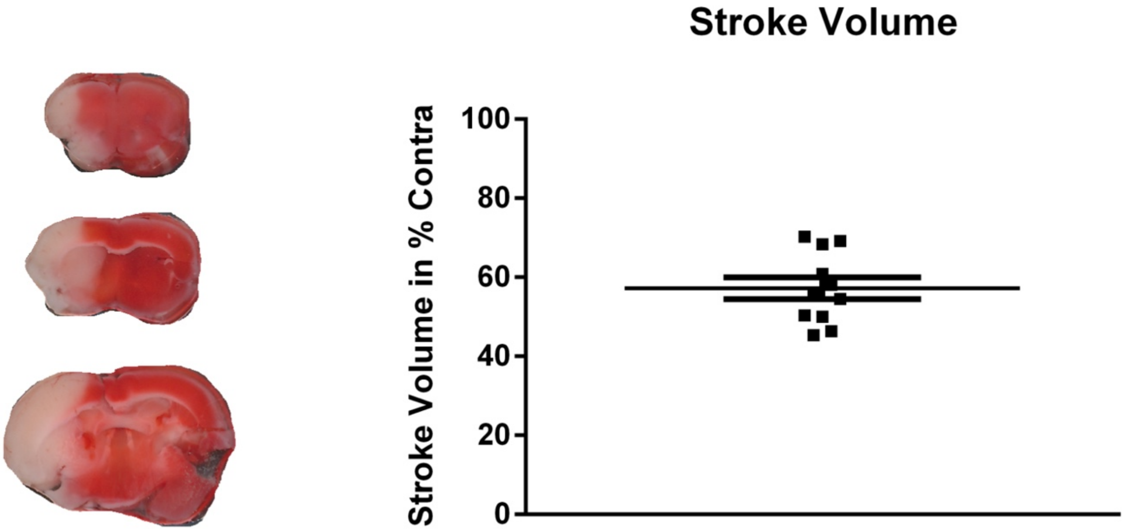

Stroke Volume Variance 24h post t-MCAO

Dot-plot (right) of stroke volumes 24h after 60 minutes occlusion of the right middle cerebral artery, measured semi-automatically using an ImageJ macro on TTC-stained 1mm brain slices (left). Due to potential edema formation in the ipsilateral hemisphere, stroke sizes are shown as area percentage of the contralateral hemisphere (mean with SEM, n = 11).
